# ﻿*Protogygiapryorensis* Crabo & Kirst (Insecta, Lepidoptera, Noctuidae, Noctuinae, Noctuini), a new moth species from Montana, United States of America

**DOI:** 10.3897/zookeys.1175.107619

**Published:** 2023-08-18

**Authors:** Lars G. Crabo, Marian L. Kirst

**Affiliations:** 1 Adjunct Faculty, Washington State University, Pullman, Washington, USA Washington State University Pullman United States of America; 2 Northern Rockies Research and Educational Services, Inc., Lolo, Montana, USA Northern Rockies Research and Educational Services Lolo United States of America

**Keywords:** Agrotina, Montana endemic, relict species, sand habitat, Wyoming Basin ecoregion

## Abstract

*Protogygiapryorensis***sp. nov.** (Insecta, Lepidoptera, Noctuidae, Noctuinae, Noctuini) is described from a single arid active sands habitat in the south foothills of the Pryor Mountains in south-central Montana, USA. It flies during early May. The adult male and its genitalia are illustrated and are compared to similar *Protogygia* McDunnough species. The female is unknown. *Protogygia* species groups are discussed and *P.pryorensis* is assigned to the *album*-group. The Pryor Mountains foothill habitat of *P.pryorensis* is described and illustrated.

## ﻿Introduction

An ongoing survey of the moths of Montana, USA—the Montana Moth Project—has been conducted by MLK and Mat Seidensticker of Northern Rockies Research and Educational Services since 2020. A series of a distinctive but unrecognized noctuid moths was collected by this group in a small area of active sands and hoodoos (weathered stone pinnacles) in Carbon County during early May, 2022. Additional specimens of this species were collected by a larger group of collectors during the same time of year in 2023. This moth, *Protogygiapryorensis* sp. nov., is described herein.

The North American noctuid genus *Protogygia* McDunnough was revised by Lafontaine and Fauske (2004). *Protogygia* contains 17 species—including *P.pryorensis*—and is assigned to Noctuinae, Noctuini, Agrotina (Pohl et al. 2016). The genus is defined in detail by Lafontaine and Fauske and its structural characters are not repeated here. *Protogygia* species are distributed in the American West from Mexico to southern Canada, mostly in the Great Plains, Intermountain, and inland West Coast regions. They are most common in open xeric habitats and many species are restricted to sand habitats, including a few that are dune obligates. *Protogygia* are medium-size moths with forewing lengths from 13 to 20 mm. The majority of the species are grayish brown or gray with a forewing pattern consisting of longitudinal streaks, with fused elongate or obscure spots and faint or absent transverse lines; however, the genus is variable, and two species are nearly pure white and a few species have a typical noctuid pattern with transverse lines and normally-shaped spots.

## ﻿Materials and methods

Wing pattern and genitalia structure terminology follow Lafontaine (1998, 2004).

Forewing lengths from base to apex, excluding the fringe, were measured to the nearest half millimeter.

Genitalia dissection techniques follow those of Hardwick (1950) and Lafontaine (2004). Detached abdomens were macerated in hot 10% potassium hydroxide for 20–30 mins. Dissections were performed in water followed by hardening in isopropyl alcohol. Vesicas were inflated. Preparations were stained with orcein (Sigma Chemical Company, St. Louis, Missouri) and mounted in Euparal (Bioquip Products, now closed permanently but formerly of Rancho Dominguez, California; hereafter Bioquip) under elevated cover glass on glass slides.

Moth sampling at Petroglyph Canyon was conducted during the potential flight season of *P.pryorensis* on May 6, 2022 (three light traps, one illuminated sheet), June 3, 2022 (three light traps, one sheet), May 8–9, 2023 (13 light traps, two sheets, one malaise trap), and May 19, 2023 (two light traps). Several similar light traps were used, all modified Robinson trap design consisting of 5-gallon (19 L) buckets with various light sources [20 W Ecolite ultraviolet bulbs (Control Zone Products Ltd, Doncaster, South Yorkshire, England) and Bioquip 12 V DC and 12 V AC ultraviolet lights with F15T8/BL bulbs] positioned between vanes over funnel tops. Ethyl acetate was used as the killing agent in all light traps. The sheets were hung vertically and were illuminated by self-ballasted mercury vapor bulbs [1000bulbs.com (Mesquite, Texas)] plus one or two ultraviolet tube lights (Bioquip). The light traps and malaise traps were deployed from dusk to dawn, while the sheets were run from dusk until 0000–0100 hrs. Trap distribution at the Petroglyph Canyon locality is described in the Distribution and ecology section, below.

All specimens collected by the Montana Moth Project were pinned and identified by Chuck Harp (CSUC).

### ﻿Repository abbreviations

**CNC** Canadian National Collection of Insects, Arachnids, and Nematodes, Ottawa, Ontario, Canada

**CSUC** Colorado State University Collection, Fort Collins, Colorado, USA

**JVC** James Vargo private research collection, Mishawaka, Indiana, USA

**LGC** Lars Crabo private research collection, Bellingham, Washington, USA

**MKC** Marian Kirst private research collection, Billings, Montana, USA

**NMNH** National Museum of Natural History, Smithsonian Institution, Washington D.C., USA

## ﻿Species account

### ﻿ Protogygia pryorensis sp. nov.

Taxon classificationAnimaliaLepidopteraNoctuidae

42325188-0ADA-55FE-B66A-1D6C082E8893

https://zoobank.org/6901711B-E110-451D-8F69-C797B36E1215

Figs 1 , 2

#### Type locality.

USA: Montana: Carbon County: Pryor Mountains, Petroglyph Canyon Natural Area, 45.0167, -108.5022, 1520 m.

#### Type material.

***Holotype*, male. USA: Montana**: Carbon County: 45.01671°N, 108.502244°W, Petroglyph C[an]y[o]n Natural Area, high desert sand dunes. Juniper, sage, pine, currant, skunk sumac. 6 May 2022, 4990’ [1520 m], uv/mv trap, leg: M. L. Kirst/I. Sommerdorf. / [Crabo genitalia slide] 665 male. CNC. ***Paratypes*.** 15 m, 0 f. **USA: Montana**: Same collection data as holotype (5 m); [same locality as holotype] Petroglyph C[an]y[o]n B[ureau of] L[and] M[anagement], 45.016, -108.502, 8–9 May, 2023, 1470 m, L. Crabo, M. Kirst, C. Harp (10 m). CNC, CSUC, JVC, LGC, MKC, NMNH.

#### Diagnosis.

**Figure 1.**
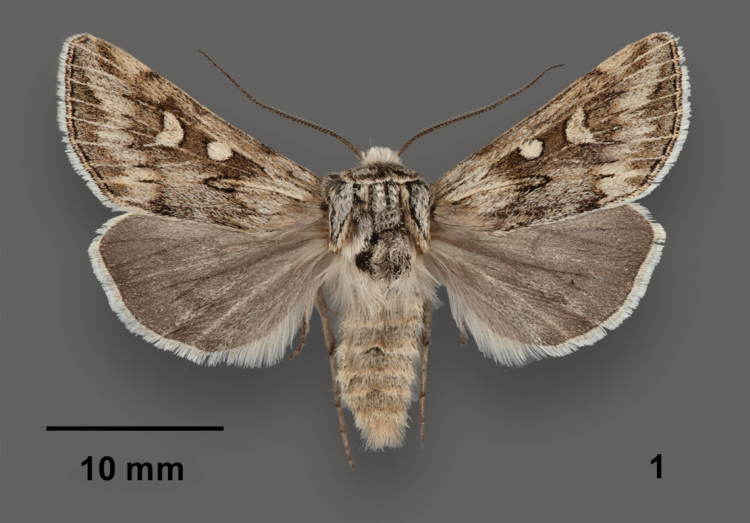
*Protogygiapryorensis*, paratype male, USA, Montana, Carbon County, Pryor Mountains, Petroglyph Canyon Natural Area.

*Protogygiapryorensis* is a superficially distinctive moth (Fig. 1). It is the only *Protogygia* species with a combination of cream-filled oval orbicular and crescentic reniform spots, sharply dentate postmedial line, and gray hindwing with white fringe. Although not as streaked as many *Protogygia* species, the combination of light veins and strongly toothed postmedial line produces a somewhat streaky pattern on the distal forewing. *Protogygiaelevata* (Smith) and *Protogygiaarena* Troubridge & Lafontaine are the most similar *Protogygia* species to *P.pryorensis* in color and pattern. Both typically have darker central areas in the forewing spots, a more typical noctuid forewing pattern with lightly scalloped lines, and lighter hindwings. Neither of these two species is known to occur in Montana, but *P.elevata* has been found as far north as southwestern Wyoming suggesting that the distributions of it and *P.pryorensis* could potentially overlap.

**Figure 2.**
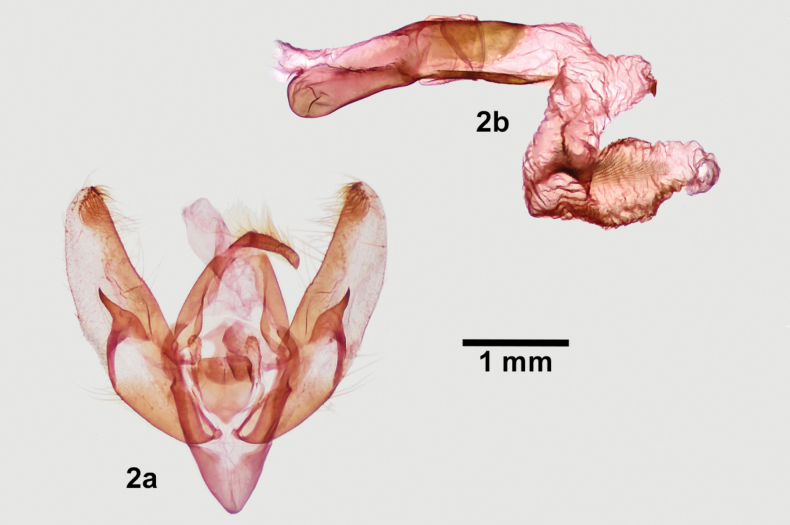
*Protogygiapryorensis*, male genitalia **a** valves **b** phallus with everted vesica.

*Protogygiapryorensis* is the only brownish *Protogygia* with a forewing pattern of complete transverse lines and distinct spots that has male genitalia with a cylindrical uncus ending in a hook and a teardrop-shaped clasper (Fig. 2a). The unci of *P.elevata* and *P.arena* are blunt tipped and swollen basally, and their claspers are apically blunted. This is discussed further in regards to species groups in the last paragraph of this section. Both of these superficially-similar species have simple hairlike scales on the dorsal thorax, which are wider and either forked and spatulate in *P.pryorensis*.

*Protogygiamilleri* (Grote) is structurally similar to *P.pryorensis* and has an unstreaked forewing with somewhat similar pattern elements (Lafontaine 2004, pl. I figs 41, 42). They key out together in couplet 12 of the “Key to Species of *Protogygia*” in Lafontaine and Fauske (2004, pp. 183, 184). Nonetheless, *P.milleri* cannot be mistaken for *P.pryorensis* because it only occurs in California and Oregon and its forewing is powdery gray rather than olive gray as in *P.pryorensis*, with simple black lines and spot outlines.

The female of *P.pryorensis* is unknown but is likely to resemble the male based on the females of other species in the genus.

Lafontaine and Fauske (2004) organize *Protogygia* into three species groups. The wing pattern of *P.pryorensis*, with unfused orbicular and reniform spots and distinct transverse lines, resembles the three species of the *elevata*-group (ibid., pl. I figs 24–28). However, *P.pryorensis* lacks the defining structural characters of this group: basal swelling and truncate apex of the uncus and truncate ampulla of the clasper (ibid., pl. 27 figs 1, 2). We assign *P.pryorensis* to the *album*-group based on its cylindrical apically hooked uncus and teardrop-shaped clasper, both typical of the other six species in this group (ibid., pl. 27 figs 3–8). All other species in the *album*-group except *P.milleri* are either nearly white or streaked longitudinally (ibid., pl. I figs 29–42).

#### Description.

**Adult male. *Head*** – Antenna biserrate and bifasciculate, width 2× central shaft; dorsal scales yellow gray; scape white. Labial palpus scales cream and scattered gray, additional long dark gray hairlike scales on sides; haustellum well developed. Frons bulging; scales of frons and vertex hairlike, long, cream, with scattered medium gray scales near eyes and between antennae. Eye normal size, hairless, with long dark posterior lashes. ***Thorax*** – Dorsal vestiture long, narrow, forked, or weakly spatulate serrate, cream, ochre, and black scales, appearing hoary light gray with faint dark pattern, a diffuse darker gray line across prothoracic collar and dark tufts in some specimens; tegula with diffuse black V and gray-tan or tan medial margin; short loose dorsal tufts on meso- and metathorax. ***Legs***: Prothoracic tibia with ~ 5 spinelike setae along sides, apical pair slightly longer and ~ 2× as stout as the others; mesothoracic and metathoracic tarsi with three rows of setae. ***Wings***: Forewing length 12.5–15.0 mm (*N* =16); distal wing elongated to bluntly pointed apex with smooth convex outer margin; scales straplike, serrate, mixed orange tan, cream, and black; wing base proximal to antemedial line, postmedial area, and veins olivaceous pale gray; veins bordered by thin black lines in medial and terminal areas; medial and subterminal areas medium dark olive gray, posterior half of medial area frosted with light gray; transverse lines dark gray, single, diffuse except as noted; basal and medial lines absent; antemedial line indistinct, convex laterally thrice, strongest on mid-wing, distal side variably shaded dark gray to black; postmedial line strongly dentate, diffuse except for long thin teeth on veins, proximal shade similar to distal shade of antemedial line; subterminal line jagged, diffuse dark brown with intervenal smudgy dark wedges; terminal line thin, black; fringe mostly cream with light tan base and thin medial line; spot outlines thin, black; claviform spot complete or incomplete, thicker than other spots, filled with adjacent ground; orbicular spot ovoid, size variable, filling light cream; reniform spot strongly kidney-shaped to crescentic, filling darker cream, rarely with light gray center. Hindwing medium gray with slightly darker veins, diffuse discal spot, and diffuse postmedial line; fringe white with cream base and thin light gray medial line. ***Abdomen*** – Lacking structural modifications such as hair pencils or pockets; vestiture uniform grayish cream. ***Male genitalia***: (Fig. 2) Uncus cylindrical, width nearly uniform, tapering to slight apical downward hook. Juxta shield shaped, height 1× width. Clavus knoblike with apical short setae. Valve straplike, length ~ 4× width, widest at mid-sacculus due to broad ventral bulge, ventral process absent, tapering slightly distally to unmodified blunt apex bearing small corona of loose clawlike setae; sacculus length ½ × valve length, nearly reaching dorsal valve near base; clasper ampulla on mid-valve, teardrop shaped with broad base tapering to acute apex. Phallus tubular, length 4× width; vesica base bent 90° rightward, then coiled 360°, widest near apex; relatively short fingerlike subbasal diverticulum with apical short acute cornutus. ***Female genitalia***: Unknown.

#### Etymology.

The species epithet pays homage to the ecologically rich Pryor Mountains in south-central Montana, where MLK as a child spent many happy hours exploring. The Pryor Mountains are named for Nathaniel Hale Pryor, a Sergeant with the Lewis and Clark Expedition that traveled through Montana in 1805.

#### Distribution and ecology.

The only known locality for *P.pryorensis* near the north mouth of Petroglyph Canyon is located in the Petroglyph Canyon Natural Area, a 97-ha system of high-desert bluffs and drainages in the southern foothills of Big Pryor Mountain in southern Carbon County, Montana. This area is in the most northern portion of the Wyoming Basin Ecoregion, a region centered in western Wyoming that barely extends into southern Montana (United States Environmental Protection Agency 2000). The Pryor Mountains are unglaciated, unique for a Montana range, and are instead the erosional product of uplifted and tilted sedimentary blocks: a western block (Big Pryor Mountain) and an eastern block (East Pryor Mountain). The range rises abruptly from the surrounding prairie to an elevation of more than 2400 m, resulting in essentially a sky island that is unique within Montana (Anonymous 2023). These mountains are notable for their unique geology, animal diversity, and globally rare plant communities that include regionally-endemic plant species such as Shoshonea [*Shoshoneapulvinata*, Evert & Constance (Apiaceae)] and thick-leaf bladderpod [*Physariapachyphylla* O’Kane & Grady (Brassicaceae)] (Lyman et al. 2015). The foothills southwest of Big Pryor Mountain contains Montana’s only true red desert and receive less than 13 cm of annual rainfall due to the double rain shadow of the Beartooth Mountains to the west and the Pryor Mountains to the north (Anonymous 2023). Petroglyph Canyon runs northwest to southeast for 1.5 km. (Anonymous 2015). Its northern trailhead is at a contact between the Kootenai (Cretaceous), Fall River, and Thermopolis Formations. These formations are characterized by sedimentary deposits of varying hardness, including thick shale beds, bentonitic mudstones, fine-grained and course-grained sandstones, and conglomerates (Lopez 2000).

**Figures 3, 4.**
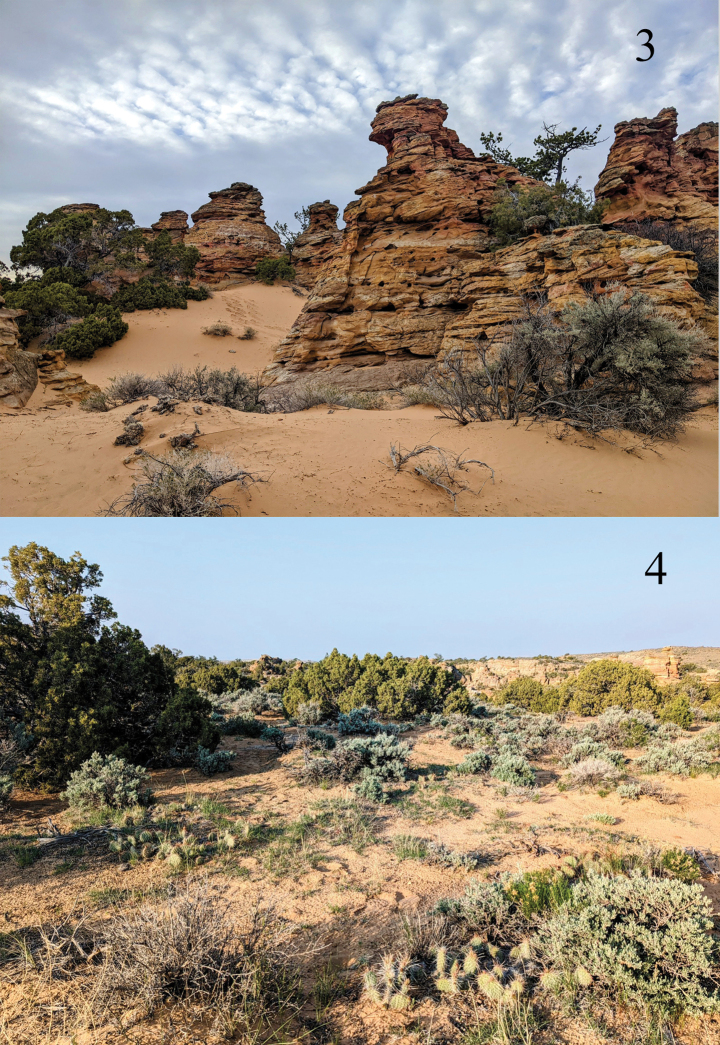
*Protogygiapryorensis* habitat at Petroglyph Canyon Natural Area, Carbon County, Montana, USA **3** shows active sands and sandstone hoodoos at a canyon entry, while **4** is a nearby area of mixed soils and interspersed bedrock above the canyon rim. Sparse woody vegetation of *Juniperusosteosperma*, *Pinusflexilis*, *Chrysothamnosviscidiflorus* and *Artemisiatridentata* and mats of *Opuntiapolyacantha* are depicted.

The *P.pryorensis* type locality (Figs 3, 4) lies fully within the Kootenai (Cretaceous) formation. It is a relatively small patch of active sands and coppice dunes surrounding spirelike hoodoos composed of cross-bedded fluvial sandstone (M. Smith pers. comm. January, 2023). It is sparsely vegetated with trees, shrubs, and herbaceous plants. The predominant trees are limber pine [*Pinusflexilis* James (Pinaceae)] and Utah juniper [*Juniperusosteosperma* (Torr.) Little (Cupressaceae)], with shrubs consisting primarily of big sage [*Artemisiatridentata* Nutt. (Asteraceae)], green rabbitbrush [*Chrysothamnosviscidiflorus* Hook. (Nutt.) (Asteraceae)], currant [*Ribes* spp. (Grossulariaceae)], and skunkbush sumac [*Rhustrilobata* Nutt. (Anacardiaceae)]. Dominant forbs include phlox [*Phlox* spp. (Polemoniaceae)], *Cryptantha* spp. (Boraginaceae), desert dandelion [*Malacothrix* spp. (Asteraceae)], and *Streptanthellalongirostrus* S. Watson, Rydb. (Brassicaceae) (Lesica et al. 2012). Extensive low mats of prickly pear cactus [*Opuntiapolyacantha* Haw. (Cactaceae)] are present on the sand and fine-grained soils.

All *P.pryorensis* were collected within a 200 m radius (approximately 3.2 ha) centered on 45.016, -108.502, where most of the traps were placed. This area includes areas of confluent sand as well as areas of mixed sand, bedrock, and fine-grained soil. The sand habitat with which *P.pryorensis* appears to be most closely associated extends farther to the north as well as into the canyon, but these areas were sampled only with one trap placed on the canyon floor on May 8, 2023 and one placed between the canyon floor and upper sand habitat on May 19, so the local distribution of this species is probably underestimated. All specimens were collected in traps and none at sheets, suggesting either that the flight is after midnight or that *P.pryorensis* is not attracted to bright mercury vapor light. All specimens were found from May 6 to May 9, with none collected May 19 or June 1, indicating a brief flight period. All six specimens from May 6, 2022 are fresh, while a few of the specimens from May 8 and 9, 2023 demonstrate slight wear. No females have been found.

The early stages of *P.pryorensis* are unknown. It is possible that the larvae burrow in sand, a habit of known larvae from the closely-related genus *Copablepharon* Harvey (Lafontaine and Fauske 2004).

## ﻿Discussion

*Protogygiapryorensis* might occur in other desert, active sands systems in the western Great Plains, but could have been overlooked due to its specialized habitat and early flight season. It is only known from Montana, but probably also occurs in Wyoming considering that the type locality is just 2 km north of the Montana-Wyoming border in the Wyoming Basin Ecoregion. It is conceivable that *P.pryorensis* is an isolated relict species with a very restricted distribution since the Pryor Mountains are unglaciated, have an unusually xeric climate for the region, as well as a unique flora.

The Pryor Mountains and surrounding region are subject to human-use pressures, including mining, grazing, energy development, and ATV recreation (Anonymous 2023). Further study will be needed to ascertain how these activities might impact *P.pryorensis*. In particular, the moth’s distribution, phenology, population size, hostplants, and habitat preferences are relevant. Fortunately, the type locality is currently protected within a BLM-designated Area of Critical Environmental Concern because Petroglyph Canyon contains petroglyph cultural artifacts (Anonymous 2015).

## Supplementary Material

XML Treatment for Protogygia pryorensis

## ﻿Additional information

### Data availability

All of the data that support the findings of this study are available in the main text.
